# Three-Dimensional Evaluation of Treatment Effects and Post-Treatment Stability of Maxillary Molar Intrusion Using Temporary Anchorage Devices in Open Bite Malocclusion

**DOI:** 10.3390/jcm13102753

**Published:** 2024-05-07

**Authors:** Hiroki Ogura, Kento Numazaki, Toshihito Oyanagi, Masahiro Seiryu, Arata Ito, Takahiro Noguchi, Fumitoshi Ohori, Michiko Yoshida, Tomohiro Fukunaga, Hideki Kitaura, Itaru Mizoguchi

**Affiliations:** Division of Orthodontics and Dentofacial Orthopedics, Tohoku University Graduate School of Dentistry, 4-1 Seiryo-machi, Aoba-ku, Sendai 980-8575, Japan; hiroki.ogura.e4@tohoku.ac.jp (H.O.); toshihito.oyanagi.a7@tohoku.ac.jp (T.O.); masahiro.seiryu.b5@tohoku.ac.jp (M.S.); arata.ito.c7@tohoku.ac.jp (A.I.); takahiro.noguchi.d4@tohoku.ac.jp (T.N.); fumitoshi.ohori.b4@tohoku.ac.jp (F.O.); michiko.yoshida.d8@tohoku.ac.jp (M.Y.); tomohiro.fukunaga.d1@tohoku.ac.jp (T.F.); hideki.kitaura.b4@tohoku.ac.jp (H.K.); mizo@tohoku.ac.jp (I.M.)

**Keywords:** orthodontic treatment, molar intrusion, temporary anchorage devices (TADs), mini-screws, open bite, three-dimensional, post-treatment stability

## Abstract

**Background:** We investigated treatment outcomes and post-treatment stability in 10 patients with an anterior open bite and nonsurgical orthodontics. **Methods:** The patients underwent maxillary molar intrusion using temporary anchorage devices (TADs) to deepen the overbite due to mandibular autorotation. Lateral cephalograms and dental cast models were obtained before treatment (T0), immediately after it (T1), and >1 year after it (T2). Skeletal and dental cephalometric changes and three-dimensional movements of the maxillary dentitions were evaluated. **Results:** At T0, cephalometric analysis indicated that patients had skeletal class I with tendencies for a class II jaw relationship and a skeletal open bite. During active treatment (T0 to T1), the maxillary first molar intruded by 1.6 mm, the mandibular first molar extruded by 0.3 mm, the Frankfort-mandibular plane angle decreased by 1.1°, and the overbite increased by 4.1 mm. Statistically significant changes were observed in the amount of vertical movement of the maxillary first molar, Frankfort-mandibular plane angle, and overbite. Three-dimensional (3D) dental cast analysis revealed that the maxillary first and second molars intruded, whereas the anterior teeth extruded, with the second premolar as an infection point. In addition, the maxillary molar was tipped distally by 2.9° and rotated distally by 0.91°. Statistically significant changes were observed in the amount of vertical movement of the central incisor, lateral incisor, canine and first molar, and molar angulation. From T1 to T2, no significant changes in cephalometric measurements or the 3D position of the maxillary dentition were observed. The maxillary and mandibular dentitions did not significantly change during post-treatment follow-up. **Conclusions:** Maxillary molar intrusion using mini-screws is an effective treatment for open bite correction, with the achieved occlusion demonstrating 3D stability at least 1 year after treatment.

## 1. Introduction

Anterior open bite is defined as the absence of overlap of the anterior teeth during intercuspal occlusion [1]. In Japan, open bite malocclusion is found in 5.7% of children and young adults [2], leading to significant functional and aesthetic impairments. Based on cephalometric analysis, anterior open bite can be categorized into dentoalveolar and skeletal types. In the former, there is incisor infraversion and proclination and slight supraversion of the molars [3]. This type is often associated with oral habits such as finger-sucking and tongue-thrusting [4]. Conversely, the latter, also known as “long face”, is morphologically characterized by a steep mandibular plane, large anterior facial height, small mandibular ramus, and posterior facial heights, and a deep antegonial notch [5,6,7].

Anterior open bite is one of the most challenging malocclusions to correct in terms of achieving appropriate overjet and maintaining post-treatment stability [1]. There are two approaches: combined surgical-orthodontic treatment and nonsurgical orthodontic treatment [8]. In the surgical approach, LeFort I osteotomy and, if necessary, bilateral sagittal split osteotomy are applied to elevate the maxilla, rotate the mandible counterclockwise, and reposition the mandible anteroposteriorly to deepen the overbite [8,9,10]. Although this approach can improve the bite without requiring a large compensatory tooth movement, there are concerns regarding post-treatment stability [9,10].

In the orthodontic approach, multibracket appliances, such as edgewise appliances, are commonly used to reposition the permanent teeth to appropriate positions. These appliances consist of brackets, archwires, and ligation wires. The brackets contain a rectangular-shaped slot and are affixed to the teeth. The archwires are ligated into the bracket slots and transmit orthodontic force due to their elasticity. In the treatment of anterior open bite, edgewise appliances are employed to extrude the incisors or intrude the molars. However, conventional treatment using edgewise appliances combined with adjunct appliances, such as vertical elastics and headgear, is associated with a high relapse rate during the retention phase [11,12]. Intruding molars using only conventional orthodontic appliances is challenging. However, the recent development of temporary anchorage devices (TADs), including mini-screws and plates, has revolutionized orthodontic treatment by providing powerful anchorage and a noncompliant approach, enabling efficient molar intrusion [13,14,15,16,17,18,19,20]. Mini-screws are small screw-shaped implants made of titanium alloy, designed for temporary placement in the alveolar bone. These screws consist of a screw part, measuring 6–10 mm in length and 1.3–2.0 mm in diameter at the thickest part, and an external head with ligature holes. The screw part is inserted into the alveolar bone. Orthodontic materials such as elastic modules, metal ligature wires, and metal coil springs can be engaged in the head to transmit orthodontic forces to the teeth. Molar intrusion facilitates mandibular autorotation, resulting in closure of the open bite, reduction of the anterior facial height, and advancement of the retruded chin. Although previous studies have demonstrated that treatment with TADs achieves satisfactory dental and skeletal improvements comparable to surgical orthodontic treatment [13,14,15,16,17,18,19,20], concerns regarding post-treatment stability have not been adequately addressed. 

We evaluated the cephalometric effects of treatment and post-treatment relapse in patients with an anterior open bite following treatment involving maxillary molar intrusion using mini-screws. Furthermore, we investigated the three-dimensional (3D) movement of the maxillary teeth using digital dental casts. In recent years, cone beam computed tomography (CBCT) and digital dental cast are widely used for diagnosis and treatment outcome assessments in malocclusions, which are powerful approaches to obtain three-dimensional (3D) information [21]. In the present study, we evaluated not only treatment and posttreatment outcomes using conventional cephalometric analysis, but also 3D tooth movement using digital dental casts. The digital dental models can provide precise 3D data on movement of a given tooth, which is difficult in cephalometric analysis.

## 2. Materials and Methods

### 2.1. Ethical Statement and Study Design

The protocol for this retrospective study was approved by the Ethics Committee of Tohoku University Hospital (no. 22183). This study included patients treated at the Tohoku University Hospital between April 2008 and March 2020. We enrolled patients with a pretreatment anterior overbite < 0 mm; Angle class I or II malocclusion; minimal remaining growth of the jaws during treatment; no previous orthodontic treatment; no congenital craniofacial anomalies, such as cleft lip and palate, syndrome, or systemic diseases; no drug use; no signs of degenerative disease of the temporomandibular joint; no missing teeth except for the third molars; skeletal maturation as evaluated by a statural height record and if needed, a hand-wrist radiograph; and available cephalometric analyses with adequate image quality at pretreatment, immediate post-treatment, and >1 year post-treatment. 

The sample size was determined based on a previous study [22], in which the difference between the means of U6-palatal plane at two different time points and the mean of the standard deviations were set at 2.6 and 2.19, respectively. With a significant difference threshold and power set at 0.05% and 80%, respectively, the required sample size was calculated to be 9 using a decision table based on paired *t*-test. Finally, we enrolled one male and nine female patients.

### 2.2. Patient Characteristics

The mean age of the patients was 25.7 (range: 14.3–52.8) years before treatment (T0), 29.2 years immediately after treatment (T1), and 30.2 years at 1-year post-treatment (T2). Four patients were treated without extraction, except for the third molars. The maxillary and mandibular bilateral premolars were extracted in four patients, whereas only maxillary premolars were extracted in two patients. 

### 2.3. Orthodontic Procedures

**Figure 1:**
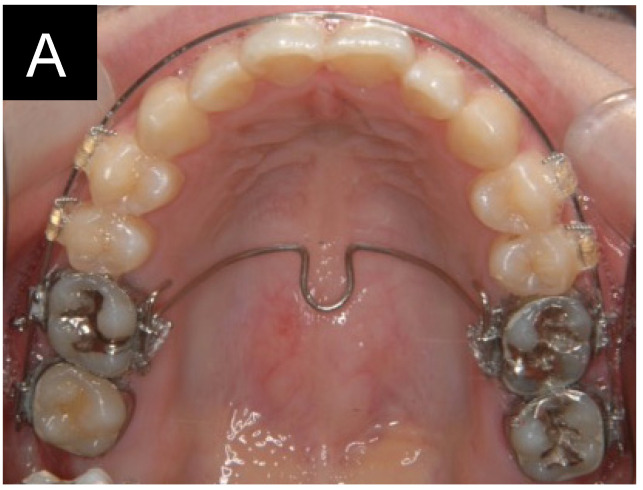

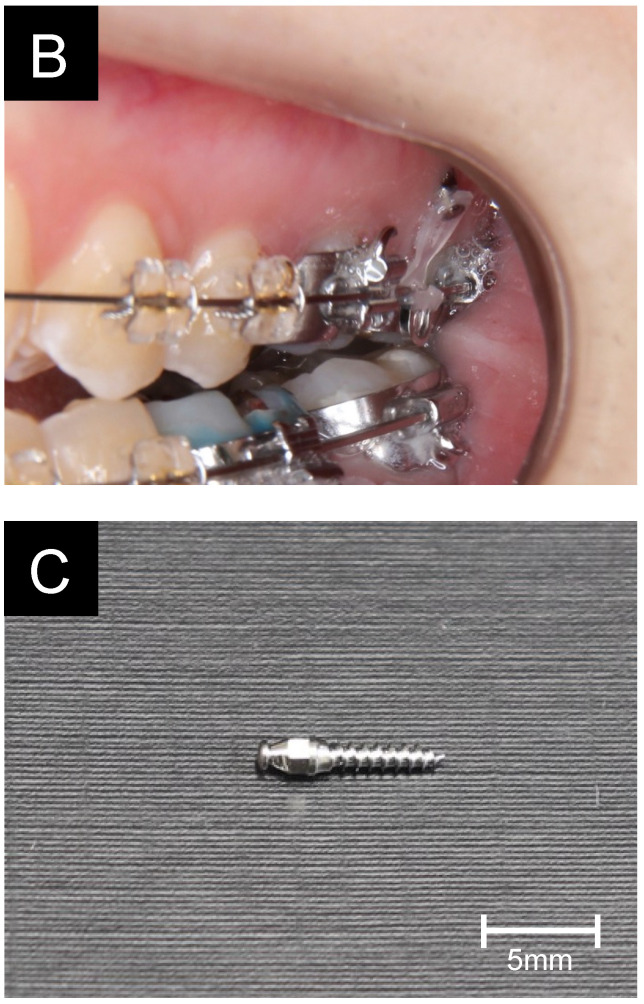
Mechanics for intrusion of the maxillary molars. (**A**) occlusal view. (**B**) lateral view. (**C**) mini-screw.

All patients were treated with a preadjusted edgewise appliance using brackets with a nominal slot size of 0.018 in (Crystabrace 7; Dentsply, Tokyo, Japan). Mini-screws (AbsoAnchor, Dentos Inc., Daegu, Republic of Korea; 1.3 mm in diameter, 5.0–7.0 mm in length) were placed in the buccal interdental regions between the first and second molars or the second premolar and first molar (Figure 1). Since interdental space and cortical bone thickness are major factors for the stability of mini-screws [16,23], the placement sites of the mini-screws were determined based on these two factors measured from medical CT images. Fourteen mini-screws were placed in the buccal alveolar bone between the maxillary first and second molars. Six mini-screws were placed between the second premolar and the first molar. A continuous archwire (0.016 × 0.022-inch stainless steel) was placed in the posterior teeth, and a transpalatal arch was placed on the maxillary first molars. Elastics were engaged between the mini-screws and the continuous archwire to intrude the maxillary posterior teeth. The intrusion force generated by the elastics was approximately 200 g per side. The mean treatment period of molar intrusion was 7.1 months, and subsequent treatment, including the alignment of the maxillary anterior teeth, was continued. The duration of active treatment was 3.1 ± 0.68 years.

### 2.4. Cephalometric Analysis

**Figure 2:**
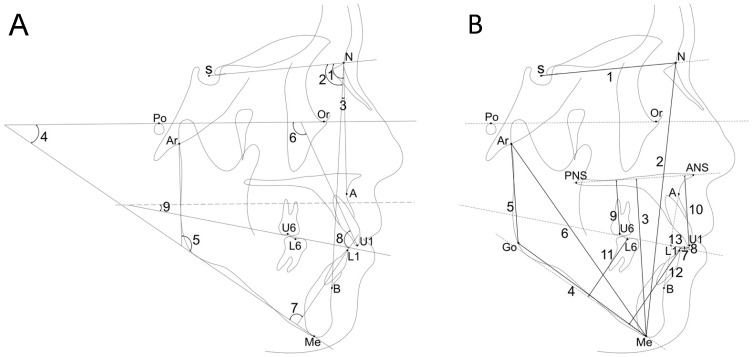
Angular and linear cephalometric measurements. (**A**) Angular measurements: 1, SNA; 2, SNB; 3, ANB; 4, MP-FH angle; 5, Go. A.; 6, U1-FH; 7, L1-MP; 8, interincisal angle (IIA); 9, OP-FH. (**B**) Linear measurements: 1, S-N; 2, N-Me; 3, Me/PP; 4, Go-Me; 5, Ar-Go; 6, Ar-Me; 7, overjet; 8, overbite; 9, U6/PP; 10, U1/PP; 11, L6/MP; 12, L1/MP;13, Wits appraisal.

Cephalograms obtained at each time point were analyzed for parameters related to skeletal and dental structures [24]. The cephalometric landmarks, reference planes, and measurement variables are presented in Figure 2.

(1) Cephalometric landmarks

Nasion (N): the most anterior point of the nasomaxillary suture; Sella (S): the geometric center of the sella turcica of the sphenoid bone; Orbitale (Or): the lowest point on the inferior orbital margin; Porion (Po): the most superior point of the outline of the external auditory meatus; Articulare (Ar): intersection of the posterior contour of the condyle and the posterior cranial base; Anterior nasal spine (ANS): the most anterior point on the bony hard palate; Posterior nasal spine (PNS): the most posterior point on the bony hard palate; Point A (A): the deepest point of the maxillary basal arch; Upper 1 (U1): incisal edge of the maxillary central incisor; Lower 1 (L1): incisal edge of the mandibular central incisor; Upper 6 (U6): midpoint of the occlusal surface of the maxillary first molar; Lower 6 (L6): midpoint of the occlusal surface of the mandibular first molar; Point B (B): the deepest point of the mandibular basal arch; Gonion (Go): the most posterior and inferior point on the outline of the angle of the mandible; Menton (Me): the most inferior point on the mandibular symphyseal outline.

(2) Reference planes

Sella-nasion (SN) plane: line connecting S to N; Frankfort horizontal (FH) plane: line connecting Or to Po; Palatal plane (PP); line connecting ANS to PNS; Mandibular plane (MP): line passing through Me tangentially to the inferior contour of the gonial angle; mandibular ramus plane; line passing through Ar tangentially to the posterior contour of the gonial angle.

(3) Angular cephalometric measurements

SNA: angle between the intersection of SN and NA; SNB: angle between the intersection of SN and NA; ANB: angle between the intersection of NA and NB; Mandibular plane (MP-FH) angle: angle between the MP and FH planes; Gonial angle (Go. A.): angle between the ramus plane and mandibular plane; U1-FH angle: angle between the long axis of the maxillary central incisor and FH plane; L1-MP angle: angle between the long axis of the mandibular central incisor and MP; interincisal angle (IIA): angle between long axes of the maxillary and mandibular central incisors; Occlusal plane angle (OP-FH): angle between the occlusal plane, which passes through the midpoint of U6 and L6, midpoint of U1 and L1, and FH plane.

(4) Linear cephalometric measurements

Anterior cranial base length: distance between S and N; Anterior facial height: distance between N and Me; Anterior lower facial height: distance between Me and PP; Mandibular body length: distance between Go and Me; Mandibular ramus length: distance between Ar and Go; Total mandibular length: distance between Ar and Me; overjet: overbite; Maxillary molar height: distance between U6 and PP; Maxillary incisor height: distance between U1 and PP; Mandibular molar height: distance between L6 and MP; Mandibular incisor height: distance between L1 and MP; Wits appraisal: distance between two points of intersection of the perpendicular lines from points A and B to the occlusal plane.

### 2.5. Dental Cast Analysis

To analyze the 3D tooth movement of the maxillary teeth, dental cast models were obtained at each timepoint. A 3D surface scanning system (Rexcan DS2′ Solutionix, Seoul, Republic of Korea), connected to a personal computer, was utilized to acquire 3D data of the dental cast model, as described previously [25]. The acquired data were saved in STL format and superimposed using a 3D analysis software (Geomagic Qualify 2013; 3D Systems, Cary, NC, USA).

**Figure 3:**
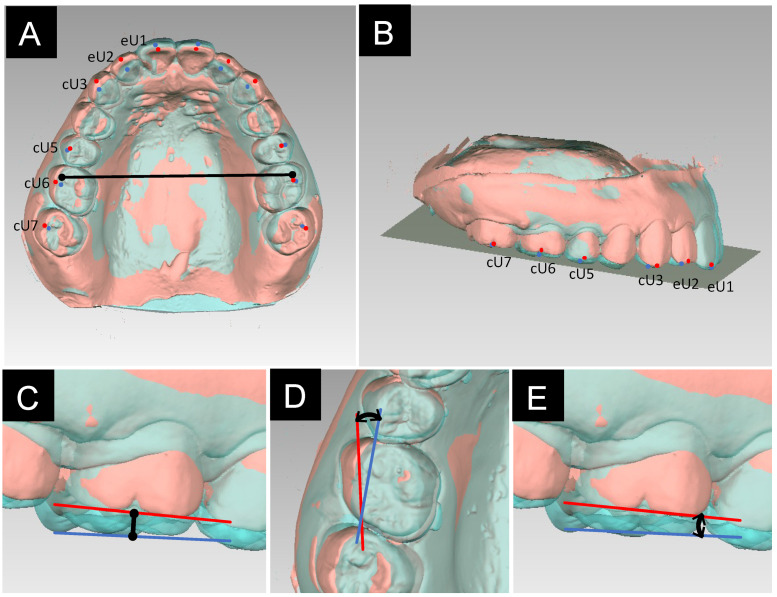
Dental cast analysis. (**A**,**B**) Anatomical landmarks and the reference occlusal plane of the maxillary dentition at T0 and T1. (**C**) Tooth movement during T0–1. (**D**) Molar angulation during T0–T1. (**E**) Molar rotation during T0 and T1. eU1, midpoint of the incisal edge of the maxillary central incisor; eU2, midpoint of the incisal edge of the maxillary lateral incisor; cU3, cuspid of the maxillary canine; cU5, buccal cusp of the maxillary second premolar; cU6, midpoint between the mesiobuccal and distobuccal cusps of the maxillary first molar; cU7, midpoint between the mesiobuccal and distobuccal cusps of the maxillary second molar. Red point and line, T0; blue point and line, T1.

Anatomical landmarks of the maxillary teeth were placed on the T0–2 dental models as follows (Figure 3A): 

eU1: Midpoint of the incisal edge of the maxillary central incisor.

eU2: Midpoint of the incisal edge of the maxillary lateral incisor.

cU3: Cuspid of the maxillary canine.

cU5: Buccal cusp of the maxillary second premolar.

cU6: Midpoint between the mesiobuccal and distobuccal cusps of the maxillary first molar.

cU7: Midpoint between the mesiobuccal and distobuccal cusps of the maxillary second molar.

The occlusal plane of the dental models at T0, which passed from the midpoint of the right and left eU1 points of the central incisors to the right and left cU6 points of the first molars, served as the reference plane for dental cast analysis (Figure 3B). The T1 and T2 models were superimposed onto the T0 model. The region of superimposition was selected based on the medial point of the third palatal rugae and the shape of the palatal vault [25,26]. 

Vertical tooth movement of maxillary teeth, intermolar width, and rotation and angulation of the maxillary first molars were evaluated as follows:

(1) Degree of vertical tooth movement: Linear distance between corresponding dental landmarks perpendicular to the occlusal plane (Figure 3C). Intrusion and elongation are denoted by positive and negative values, respectively.
(2) Intermolar width: Distance between the mesiobuccal cusps of the left and right molars (Figure 3A).
(3) Degree of molar rotation: Changes in the angulation between the lines connecting the mesiobuccal and distobuccal cusps of the first molar at the two time points (Figure 3D). Mesial and distal rotations are denoted by positive and negative values, respectively.
(4) Degree of molar angulation: Changes in the angulation between the lines connecting the mesiobuccal and distobuccal cusps of the first molar at the two time points (Figure 3E). Mesial and distal tipping are denoted by positive and negative values, respectively.

### 2.6. Error Analysis

To ensure the reliability of measurements, a single investigator (HO) performed repeated measurements at an interval of 2 weeks, and the mean with standard deviation was recorded. Error was determined using Dahlberg’s formula, SE = √(Σd2/2n), where n = 10 and d = the difference between two measurements. The reliability of the data was evaluated based on the intraclass correlation coefficient (ICC).

### 2.7. Statistical Analysis

JMP Pro software (version 17.0.0; SAS Institute Inc., Cary, NC, USA) was used for statistical analyses. Cephalometric measurements with a normal distribution were analyzed using the paired *t*-test, and Wilcoxon’s signed-rank test was used to analyze data with a non-normal distribution. *p*-values < 0.05 were considered indicative of statistical significance.

## 3. Results

### 3.1. Method Errors and Intraobserver Reproducibility

The method error of cephalometric analysis ranged from 0.105 to 0.459 mm for linear measurements and from 0.164° to 0.407° for angular measurements. The method error of cast model analysis ranged from 0.045 to 0.355 mm. These results indicate that the present analysis is more reliable than previous studies with technical errors [16,19,22]. Intraobserver reproducibility was evaluated based on the ICC, which ranged from 0.984 to 0.998 for all cephalometric variables and from 0.978 to 0.999 for the digital dental model assessment.

### 3.2. Cephalometric Analysis

Cephalometric analysis revealed that patients exhibited skeletal class I with tendencies toward class II (ANB of 5.7°) at T0 (Table 1). Furthermore, there was a tendency toward skeletal open bite (high angle), although the MP-FH angle (33.8°) was within one standard deviation of the reference value. The overjet and overbite were 5.6 and –2.4 mm, respectively. 

From T0 to T1, the U6/FH decreased by 1.5 mm, whereas the L6/MP increased by 0.5 mm. U1/FH and L1/MP increased by 1.1 and 0.3 mm, respectively. The ANB angle decreased by 0.5°, and the MP-FH angle decreased by 1.1°. Conversely, dental, and skeletal measurements remained largely unchanged from T1 to T2. Statistically significant changes were observed in MP-FH, U1-FH, L1-MP, IIA, OP-FH, N-Me, Me/PP, overjet, overbite, U6/FH, U1/FH, and Wits from T0 to T1.

### 3.3. Dental Cast Analysis

**Figure 4:**
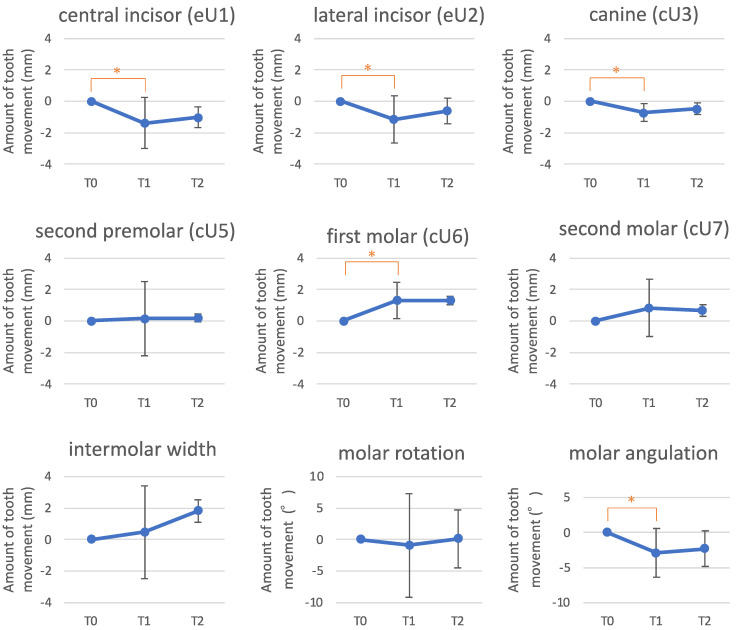
Vertical tooth movement of the maxillary teeth and changes in intermolar width and maxillary first molar rotation and angulation. T0, pretreatment; T1, post-treatment; T2, 1-year post-treatment. * *p* < 0.05.

The maxillary central incisor, lateral incisor, and canine extruded by 1.38, 1.14, and 0.72 mm, respectively, from T0 to T1 (Figure 4). The second premolar exhibited minimal vertical movement. The maxillary first molar intruded by 1.30 mm, tipped back by 2.9°, and rotated distally by 0.91°. The second molar intruded by 0.83 mm. Statistically significant changes were observed in the amount of vertical movement of the central incisor (eU1), lateral incisor (eU2), canine (cU3), and first molar (cU6), and molar angulation from T0 to T1. There was a slight relapse in the vertical position of the maxillary teeth from T1 to T2, characterized by the intrusion of the anterior teeth and extrusion of the molars, although these changes were not statistically significant. 

## 4. Discussion

Based on cephalometric measurements at T0, the patients exhibited a significant overjet, overbite, and lower facial height and mandibular molar height, whereas all other measurements were within the normal range. These findings indicated that the patients had skeletal class I with tendencies for class II malocclusion and a high-angle facial profile. Previous studies that have investigated the effects of molar intrusion with TADs [18,19,20,21,27,28,29,30,31,32] revealed an SN-MP angle at T0 ranging from 36.1° [28] to 46.5° [22]. In this study, the patients exhibited an SN-MP angle of 40.6° and an FH-MP angle of 33.9°. Notably, the study participants did not have a significant high angle deviation, potentially because patients with degenerative joint disease were excluded from the study.

This study had an unbalanced sex ratio of one male and nine females. Akbaydogan et al. [32] found no statistically significant differences between male and female patients in cephalometric parameters related to the treatment outcomes of mini-screws. Therefore, the sex ratio in this study may not have influenced the treatment outcomes.

With regard to the treatment outcomes of molar intrusion using TADs, Kuroda et al. [16] applied miniplates on the maxilla and mandible, demonstrating that the maxillary and mandibular molars were intruded by 2.3 and 1.3 mm, respectively, with an increase in the overbite of 6.8 mm. Similarly, Deguchi et al. [19] used mini-screws and found maxillary molar intrusion of 2.3 mm and increase in the overbite of 6.2 mm. Scheffler et al. [29], Foot et al. [28], and Akan et al. [31] revealed that the maxillary molars were intruded by 2.3, 2.9, and 2.3 mm, respectively, and the overbite increased by 1.2, 3.0, and 3.2 mm, respectively. These studies demonstrate no significant differences in treatment outcomes between mini-screw anchorage and miniplate anchorage. Molar intrusion leads to mandibular autorotation, which results in an increase in the overbite and ANB angle as well as a decrease in the overjet and MP angle [16,19,28,29,31]. In our study, overbite and ANB increased by 4.1 mm and 0.5°, respectively, and overjet and SN-MP decreased by 2.4 mm and 1.1°, respectively. We observed smaller treatment effects compared to previous studies, potentially because the pretreatment degree of negative overbite was not severe.

Compensatory extrusion of the mandibular molars can occur during maxillary molar intrusion [27]. Previous studies that did not conduct mandibular molar intrusion have reported extrusions of the mandibular molars of 0.1 to 1.0 mm [28,29,31]. Conversely, when using TADs, intrusion is approximately 1 mm [13,16,19]. Although the degree of mandibular molar extrusion in the present study was small (i.e., 0.3 mm), the mandibular molars should be maintained at their original vertical position, or if necessary, intruded.

There was a possibility that the mode of tooth movement was different between the extraction and non-extraction groups. We have examined whether there was a difference in the maxillary teeth that existed between the two groups, although the sample size was small. The cephalometric and dental cast analyses indicated that there was no significant difference between the two groups, although the maxillary central incisors in the extraction cases tended to move more palatally than in the non-extraction cases. However, given the small sample size, this may be attributable to a lack of power.

In the present study, all the mini-screws were placed in the buccal alveolar bone. When the intrusive force was applied to the molars from the buccally placed mini-screws, the maxillary molars cause buccal tipping. The transpalatal arch was used to prevent this side effect. Three patients were treated with the mini-screws placed in the buccal interdental regions between the second premolar and the first molar, and seven patients were treated with the mini-screws placed between the first and second molars. We examined whether there was a difference in tooth movement that existed between the two placement sites. The cephalometric and dental cast analyses indicated that there was no significant difference in the mode of tooth movement, although it was inconclusive due to the small sample size.

A recent meta-analysis showed that jaw of placement, age, screw length, and screw diameter are critical factors to the success of mini-screws [33,34]. Motoyoshi et al. [33] reported that the success rate was 63.8% in the adolescent patients under 15 years old and 91.9% in the patients over 15 years old. However, the success rate of the adolescents could be improved by a latent period of 3 months before loading [33]. All the patients used in the present study were over 15 years old. Further studies are needed to clarify whether the difference in treatment effects exists between late-adolescent and adult patients.

Elongated incisors are unstable and relapse during retention [12]. In the present study, maxillary and mandibular incisors extruded by 1.1 and 0.3 mm, respectively, during treatment. Kuroda et al. [16] reported maxillary and mandibular incisor extrusion of 0.5 and 0.1 mm, respectively, suggesting that extrusion of the incisors may occur at the time of leveling and intermaxillary elastic use during treatment [16]. One plausible explanation for incisor extrusion is palatal tipping of the maxillary incisors, particularly in cases involving extractions, which may result in apparent incisor extrusion by causing downward movement of the incisal edges. In addition, true incisor extrusion can occur due to flattening of the maxillary occlusal plane. In patients with an open bite, the occlusal planes can be convex, diverging from the premolars anteriorly, or flat, diverging from the molars anteriorly. In patients with a convex occlusal plane, full-arch leveling by a continuous arch wire can cause incisor extrusion. In comparison, in patients with a flat occlusal plane, intrusion of maxillary molars with a continuous arch can lead to clockwise rotation of the occlusal plane and extrusion of the incisors. Therefore, segmented arch leveling and intrusion of the maxillary posterior teeth are necessary to prevent incisor extrusion.

One of the challenges of the treatment of an open bite is post-treatment stability, particularly the relapse of intruded molars. Deguchi et al. [19] observed extrusion of the maxillary molars of 0.5 mm and extrusion of the mandibular molars of 1.7 mm during 2 years of post-treatment follow-up. Scheffler et al. [29] observed 0.5 mm of extrusion of the maxillary molars and 0.6 mm of intrusion of the mandibular molars during 1 year of follow-up. Marzouk et al. [35] reported 0.3 mm of extrusion of the maxillary molars and 0.3 mm of intrusion of the mandibular molars during 1 year of follow-up. Reported relapse rates for maxillary molars range from 10.2% to 23.8%, with most relapses occurring 1 year after treatment [18,19,29,30]. In our study, the maxillary molars relapsed by 0.1 mm in 6.4% of cases, whereas the mandibular molars did not relapse, indicating a low relapse rate. Although the reason for the low relapse rate for molars is unclear, it might be related to the degree of vertical skeletal discrepancies and masticatory muscle activities of the patients, extraction status of the teeth, degree of molar intrusion, and retention method [36,37]. 

The effects of orthodontic treatment and stability of the anterior overbite treatment using mini-screws were equivalent to surgical treatment [16]. Orthodontic treatment has several advantages over surgery, including less invasiveness and lower morbidity [20]. However, intrusion of molars can lead to anteriorization of the mandibular teeth relative to the maxillary teeth, resulting in a shift from a class I to class III occlusal relationship due to mandibular autorotation. In our study, the Wits appraisal decreased by 2.7 mm from T0 to T1. In cases where a significant shift to a class III occlusal relationship is anticipated, application of orthognathic surgery is appropriate. Notably, our study only included individuals with a mild or moderate anterior open bite and a class II occlusal relationship with a tendency toward a class II relationship. Therefore, the effectiveness of orthodontic treatment with mini-screws in patients with severe skeletal open bite malocclusions remains unclear. Further studies are needed to determine the limitations and long-term stability of orthodontic treatment using mini-screws.

Although the goal of orthodontic treatment is to improve oral health conditions and facial esthetics, orthodontic treatment is associated with various iatrogenic adverse effects, such as root resorption, periodontal disease, caries, and pain [38]. Orthodontists should be aware of these adverse effects and associated risk factors because until now adverse effects associated with open bite correction through molar intrusion are unclear.

## 5. Conclusions

Orthodontic treatment with intrusion of the maxillary molars using mini-screws is effective for the management of patients with a mild to moderate anterior open bite and achieves post-treatment stability. Furthermore, 3D analysis of tooth movements during treatment of an open bite can facilitate the accurate evaluation of orthodontic tooth movement and appropriate treatment planning for post-treatment stability, considering that the mode of tooth movement varies between individual patients.

## Figures and Tables

**Table 1:** Cephalometric measurements at pretreatment (T0), post-treatment (T1), and one- year posttreatment (T2).

	T0	T1	T2	T0–T1		T1–T2	
	Mean ± SD	Mean ± SD	Mean ± SD	Mean ± SD	*p* Value	Mean ± SD	*p* Value
Angular (°)							
SNA	82.7 ± 3.1	82.7 ± 3.1	82.7 ± 3.1	0.0 ± 0.0	1.000 ^a^	0.0 ± 0.0	1.000 ^a^
SNB	77.7 ± 3.0	78.2 ± 3.2	78.1 ± 3.2	0.5 ± 0.7	0.550 ^a^	−0.1 ± 0.3	0.343 ^a^
ANB	5.0 ± 1.7	4.5 ± 1.7	4.6 ± 1.7	−0.5 ± 0.7	0.589 ^a^	0.1 ± 0.2	0.193 ^a^
Mp-FH	33.8 ± 4.4	32.7 ± 4.3	32.8 ± 4.4	−1.1 ± 0.9	0.002 ^b,^**	0.1 ± 0.8	0.168 ^a^
Go A.	123.1 ± 4.8	122.9 ± 5.2	122.9 ± 5.3	−0.2 ± 1.5	0.489 ^a^	0.0 ± 0.6	0.832 ^a^
U1-FH	117.6 ± 4.4	109.3 ± 4.7	109.6 ± 4.3	−8.3 ± 6.2	0.003 ^a,^**	0.3 ± 0.5	0.372 ^a^
L1-Mp	96.3 ± 5.9	90.7 ± 4.9	90.8 ± 4.6	−5.6 ± 6.1	0.022 ^a,^*	0.1 ± 1.6	0.615 ^a^
IIA	112.2 ± 8.7	127.3 ± 7.4	126.8 ± 6.7	15.1 ± 11.0	0.004 ^b,^**	−0.5 ± 1.7	0.372 ^a^
OP-FH	19.0 ± 2.9	21.3 ± 4.0	21.6 ± 4.1	2.3 ± 1.7	0.001 ^a,^**	0.3 ± 0.8	0.059 ^a^
Linear (mm)							
S-N	65.3 ± 4.4	65.3 ± 4.4	65.3 ± 4.4	0.0 ± 0.0	1.000 ^a^	0.0 ± 0.0	1.000 ^a^
N-Me	131.8 ± 8.2	130.5 ± 8.1	130.6 ± 8.0	−1.2 ± 1.0	0.003 ^a,^**	0.1 ± 0.7	0.066 ^a^
Me/PP	74.6 ± 4.8	73.5 ± 4.7	73.6 ± 4.7	−1.1 ± 0.9	0.006 ^a,^**	0.1 ± 0.7	0.051 ^a^
Go-Me	73.1 ± 6.5	73.2 ± 7.0	73.3 ± 7.0	0.1 ± 1.2	0.277 ^a^	0.1 ± 1.1	0.111 ^a^
Ar-Go	47.6 ± 5.8	47.7 ± 5.5	47.7 ± 5.5	0.1 ± 0.7	0.938 ^a^	0.0 ± 1.0	1.000 ^a^
Ar-Me	108.6 ± 9.1	108.6 ± 9.5	108.8 ± 9.4	0.0 ± 0.9	0.664 ^a^	0.2 ± 0.7	0.528 ^a^
overjet	5.1 ± 2.5	2.7 ± 0.6	2.5 ± 0.8	−2.4 ± 2.5	0.021 ^a,^*	0.2 ± 0.5	0.282 ^a^
overbite	−2.4 ± 1.4	1.7 ± 0.7	1.6 ± 1.0	4.1 ± 1.3	0.001 ^a,^**	−0.1 ± 0.4	0.664 ^a^
U6/FH	52.2 ± 4.0	50.7 ± 3.5	50.8 ± 3.5	−1.5 ± 0.6	0.001 ^a,^**	0.1 ± 0.6	0.089 ^a^
U1/FH	57.7 ± 4.6	58.8 ± 4.2	59.0 ± 4.4	1.1 ± 1.7	0.049 ^b,^*	0.1 ± 0.4	0.219 ^b^
L6/Mp	36.3 ± 4.0	36.8 ± 4.4	36.8 ± 4.4	0.5 ± 0.8	0.129 ^a^	0.0 ± 0.4	0.685 ^a^
L1/Mp	45.7 ± 3.5	46.0 ± 3.4	45.9 ± 3.3	0.3 ± 1.3	0.485 ^a^	−0.1 ± 0.5	0.153 ^a^
Wits	0 ± 2.0	−2.7 ± 1.4	−2.9 ± 1.3	−2.7 ± 1.8	0.002 ^a,^**	−0.2 ± 1.0	0.198 ^a^

^a^ Paired *t*-test was performed. ^b^ Wilcoxon matched-pairs signed-rank test was performed. * *p* < 0.05; ** *p* < 0.01

## Data Availability

All data generated and/or analyzed in the current study are included in this article.
